# Whole-Genome Methylation Analysis of Female, Male, and Neomale Northern Pike (*Esox lucius*)

**DOI:** 10.3390/ani15243594

**Published:** 2025-12-15

**Authors:** Zhelan Wang, Qian Xiao, Jiaqing Xu, Xinan Fu, Sitong Li, Jia Wang, Junjie Zhang

**Affiliations:** 1 College of Life Sciences, Xinjiang Agricultural University, Urumqi 830052, China; m15286133601@163.com (Z.W.); qianxiao181@163.com (Q.X.); x320243911@163.com (J.X.); 18087035850@163.com (X.F.); 18160510177@163.com (S.L.); wangjia365@xjau.edu.cn (J.W.); 2 Xinjiang Key Laboratory for Ecological Adaptation and Evolution of Extreme Environment Biology, College of Life Sciences, Xinjiang Agricultural University, Urumqi 830052, China; 3 Xinjiang Characteristic Aquaculture Research Center, Urumqi 830052, China; 4 Modern Fisheries Industry College, Xinjiang Agricultural University, Urumqi 830052, China

**Keywords:** whole-genome sequencing, DNA methylation, sex determination, *Esox lucius*, neomales

## Abstract

To investigate the effect of epigenetic modifications on sex determination and differentiation in northern pike (*Esox lucius*), we employed Whole-Genome Bisulfite Sequencing (WGBS) to analyze the DNA methylation patterns in gonadal tissues of females, males, and neomales. First, we obtained high-quality sequencing data, including a total of 410.16 Gb of raw reads and 361.48 Gb of clean reads, with an 86% unique mapping rate, and a bisulfite conversion efficiency of 99.6%. Subsequently, comparative analysis revealed that 66,581 differentially methylated CG regions (i.e., DNA regions with a high frequency of CG dinucleotides), 1215 differentially methylated CHG regions (i.e., DNA regions where CG is followed by another nucleotide), and 3185 differentially methylated CHH regions (i.e., regions where cytosine is methylated in a CHH sequence, with ‘H’ representing A, T, or C) were identified among the three groups. Furthermore, we identified four key differentially methylated candidate genes (*Rspo1*, *hsd11b2*, *CYP27A1* and *smad3*) associated with sex determination and differentiation processes in *E. lucius*. Finally, by integrating GO and KEGG enrichment analyses, we explored the role of epigenetic modification regulatory networks in the sex determination and differentiation of *E. lucius* and identified multiple metabolic pathways related to sex determination and differentiation processes (Notch signaling pathway, Wnt signaling pathway and Ovarian steroidogenesis). This study thereby lays a foundation for subsequent functional verification.

## Simple Summary

Sex determination and differentiation are key biological processes of northern pike (*Esox lucius*). Whole-genome bisulfite sequencing (WGBS), which identifies relevant key genes by analyzing DNA methylation differences, has been widely used in studies on animal epigenetic regulation. However, genome-wide studies on northern pike in this field are currently scarce. This study performed WGBS on gonadal tissues of females, males, and neomales, obtaining high-quality data. It identified numerous differentially methylated regions and candidate genes, including *Rspo1*, *hsd11b2*, *CYP27A1*, and *smad3* genes, involving pathways such as Notch. This study lays a foundation for elucidating the epigenetic mechanisms underlying its sex differentiation.

## 1. Introduction

Fishes exhibit extremely high diversity in their sex determination mechanisms. Unlike mammals and birds—where sex-determining genes are concentrated on sex chromosomes—fishes harbor such genes not only on sex chromosomes but also on autosomes, with autosomal genes also widely participating in the process. Furthermore, fish sex determination not only relies on genetic factors but, in some cases, is also significantly influenced by environmental factors such as temperature and pH [1]. This dual regulation endows fishes with high plasticity in sex determination. Sex differentiation refers to the process by which undifferentiated gonads develop into testes or ovaries and depends on the regulated expression of a series of key genes: During early male differentiation, genes such as *gsdf*, *amh*, *dmrt1*, and *cyp11b* exhibit significantly high expression [2,3]; in female differentiation, genes including *cyp19a1a*, *lhcgr*, and *foxl2* play core roles [4,5]. Notably, the *dmrt1* gene acts as a core regulatory factor for testis formation and function across various vertebrates, and its expression exhibits sexual dimorphism even before the onset of sex differentiation [6,7]. Based on these characteristics of fish sex regulation, artificial manipulation can be used to breed monosex populations for aquaculture. Examples include all-male populations of Nile tilapia (*Oreochromis mossambicus*) and yellow catfish (*Pelteobagrus fulvidraco*), as well as all-female populations of Japanese flounder (*Paralichthys olivaceus*) and Chinook salmon (*Oncorhynchus tshawytscha*) [8,9,10,11].

*E. lucius* belongs to the genus *Esox*, family Esocidae, order Salmoniformes. It is distributed in freshwater basins of North America and northern Eurasia [12]; in China, it mainly inhabits the Irtysh River in the Xinjiang Uygur Autonomous Region and is one of the important indigenous economic fish species in the region [13]. It is not only a characteristic aquaculture species in Xinjiang but also a top predator in natural water bodies, capable of regulating fish population structure and influencing ecosystem stability [14]. Studies have shown that it exhibits significant sexual dimorphism in growth: females are larger in size, reach sexual maturity later, and have a longer lifespan [15]. Currently, sex-specific markers for sex identification have been identified (unpublished data); such markers have also been identified for other fish species, such as Mozambique tilapia (*O. mossambicus*) [16] and half-smooth tongue sole (*Cynoglossus semilaevis*). Although the master sex-determining gene (*amhY*) has been identified in *E. lucius* [17], the epigenetic mechanisms that fine-tune gonadal differentiation remain largely unknown. This study was therefore designed to investigate the genome-wide DNA methylation landscapes among female, male, and neomale *E. lucius*, aiming to uncover the epigenetic regulatory network underlying sex differentiation. Our findings will provide fundamental insights into the interplay between genetics and epigenetics in fish sex determination and offer valuable theoretical support for developing monosex aquaculture technologies in this economically and ecologically important species.

Whole-Genome Bisulfite Sequencing (WGBS) is a high-throughput technology used to detect DNA methylation levels across the entire genome. By treating DNA with bisulfite, WGBS can convert unmethylated cytosines to uracils, while methylated cytosines remain unchanged, thereby enabling the accurate identification of methylated sites [18]. WGBS has been widely applied in fish research, particularly to investigate environmental stress, developmental processes, disease mechanisms, and other related aspects [19,20]. As an important epigenetic mechanism regulating gene expression, DNA methylation plays a crucial role in processes such as biological development and disease occurrence, and it is particularly significant in the regulation of sex determination and differentiation. Studies have shown that changes in methylation levels can directly affect the expression of sex-related genes. This characteristic has also enabled WGBS to be widely applied in fish research, particularly in fields such as investigating environmental stress, developmental processes, disease mechanisms, and sex regulation. For example, in zebrafish (*Danio rerio*), promoter hypomethylation of *cyp19a1a* (which encodes aromatase) and promoter hypermethylation of *amh* (anti-Müllerian hormone) lead to the up-regulation of *cyp19a1a* and down-regulation of *amh*, respectively. This epigenetic regulation biases sex differentiation toward females, consistent with the findings of Yong et al., 2024 [21,22,23]. In tiger pufferfish (*Takifugu rubripes*), there are significant differences in genome-wide DNA methylation levels between females and males, as determined via WGBS analysis, particularly during gonadal development. Studies have shown that the differentially methylated regions (DMRs) between females and males are mainly distributed in structural gene and promoter regions, and these regions are closely associated with genes related to sex determination and differentiation (e.g., *amhr2* and *pfcyp19a*) [24]. Furthermore, treatment with estradiol (E2) and aromatase inhibitor (AI) significantly alters the DNA methylation patterns of the gonads in *T. rubripes*, which further confirms the importance of DNA methylation in sex regulation [25].

Collectively, these findings indicate that DNA methylation is involved in regulating the processes of sex determination, sex differentiation, and sex reversal in *E. lucius*, providing new insights into deciphering its sex regulatory mechanisms and developing sex-control technologies. Herein, we collected gonadal tissues from females, males, and neomales (sex-reversed individuals) of *E. lucius*, and performed WGBS to investigate their DNA methylation profiles. The aim of this study is to identify candidate genes and regulatory pathways associated with sex determination, sex differentiation, and sex reversal in *E. lucius*; further clarify the epigenetic regulatory patterns in the gonadal tissues of *E. lucius*; and provide a theoretical framework for *E. lucius* sex-control technologies and the development of high-quality germplasm resources.

## 2. Materials and Methods

### 2.1. Ethics Statement

All experiments performed in this study were approved by the Animal Welfare and Ethics Committee of Xinjiang Agricultural University (Urumqi, China), with the protocol code of 2022027, on 20 May 2022.

### 2.2. Fish Sample Collection

Neomales refer to individuals with female genetic sex but male phenotypic sex, while females and males are individuals with consistent genetic and phenotypic sex. All neomales, females, and males used in this study were purchased from a legitimate commercial supplier, with fish sources being wild *E. lucius* legally captured from natural water bodies. Gonadal tissues were collected from three individuals each of females, males, and neomales. A portion of the gonadal tissues was rinsed with physiological saline and fixed in 4% paraformaldehyde (Sinopharm Chemical Reagent Co., Ltd., Shanghai, China); the other portion was immediately frozen in liquid nitrogen for fixation and subsequently transferred to a −80 °C freezer for storage. Fin-clip samples were preserved in 100% ethanol (Shanghai Titan Technology Co., Ltd., Shanghai, China), and their genomic DNA was extracted using the ammonium acetate precipitation (Beijing Solarbio Science & Technology Co., Ltd., Beijing, China) method. Genetic sex of the samples was identified using unpublished sex-specific molecular markers, while phenotypic sex was verified through histological analysis of gonadal sections. Ultimately, genome-wide DNA methylation analysis was performed on gonadal tissues from three neomales, three females, and three males.

### 2.3. Sex Identification of Experimental Fish

Genomic DNA was extracted from *E. lucius* fin tissue using the ammonium acetate method. PCR amplification was performed using two pairs of laboratory-developed sex-specific primers to determine genetic sex. The primer sequences used were as follows: Amhby-F (5′-GCTCAACTTTTGTTGTTTCATTTCA-3′) and Amhby-R (AATTACCATCACAACAGCCATGC), 4-F (TCAGCCACTATATCTATCTTACCG) and 4-R (GGACTTTTTCCTACATACCTCAC). The PCR program was set as follows: 5 min of pre-denaturation at 95 °C; 36 cycles of 30 s of denaturation at 95 °C, 30 s of annealing at 58 °C, and 45 s extension at 72 °C; concluding with a 10 min final extension at 72 °C. Amplification products were analyzed by 1.5% agarose gel electrophoresis with the following interpretation criteria: Genetically female individuals amplified only a single 606-bp band using primer set 4, with no amplification products detected using the Amh primer set. Genetically male individuals amplified two bands (606 bp and 320 bp) using primer set 4, while the Amh primer set specifically amplified a 500 bp band.

Gonadal tissues were fixed in 4% paraformaldehyde at 4 °C for 24 h. Post-fixation, specimens underwent sequential processing including dehydration through a graded ethanol series, xylene clearing, and paraffin embedding. Serial sections were cut at 6 μm thickness. Following deparaffinization in xylene, sections were stained using a standardized hematoxylin and eosin (H&E) protocol. Stained slides were subsequently dehydrated through an ascending ethanol gradient, cleared in xylene, and mounted with neutral balsam and finally observed under a light microscope (Motic, Xiamen, China).

Females exhibited a genetic female (molecular marker result) and a phenotypic female (gonadal histology result); males exhibited a genetic male (molecular marker result) and a phenotypic male (gonadal histology result); neomales exhibited a genetic female (molecular marker result) and a phenotypic male (gonadal histology result).

### 2.4. Construction and Sequencing of Genome-Wide Bisulfite methylC-Seq Libraries

DNA was extracted from the gonads (frozen at −80 °C) of males, females, and neomales using the phenol/chloroform method. After passing sample quality inspection, 100 ng of genomic DNA was taken and mixed with 0.5 ng of unmethylated lambda DNA. Subsequently, the Covaris S220 ultrasonic cell disruptor (Covaris, Inc., Woburn, MA, USA) was used to shear the mixture into fragments of 200–400 bp. The 3′ ends were repaired by adding adenine with methylated linkers, followed by bisulfite treatment using the EZ DNA Methylation-Gold™ Kit (Zymo Research, Orange, CA, USA) to convert unmethylated cytosines to uracils. Subsequent steps, including adapter ligation, fragment selection, and PCR amplification, were taken to complete library construction. During this process, internal reference phage DNA (lambda DNA) was added, and the conversion rate was determined by counting phage DNA.

After library construction, quality inspection was conducted using the Agilent 5400 system (Agilent, Santa Clara, CA, USA) to evaluate library quality, and quantification was performed via PCR with the requirement that the library concentration exceeded 1.5 nM. Different libraries that passed quality inspection were pooled according to their effective concentrations and the requirements for target output data volume, and then subjected to paired-end sequencing on the Illumina sequencing platform (Illumina, San Diego, CA, USA), generating 150 bp paired-end sequencing reads.

### 2.5. Quality Control, Read Mapping, and Methylation Calling

Raw sequencing data from the Illumina platform were subjected to quality assessment using FastQC (http://www.bioinformatics.babraham.ac.uk/projects/fastqc/, accessed on 4 June 2025) and were subsequently processed with fastp (v0.23.1; https://github.com/OpenGene/fastp, accessed on 4 June 2025) to remove adapters and low-quality reads, yielding high-quality clean data. The efficacy of filtering was confirmed by a second FastQC analysis. Repetitive elements and CpG islands (CGIs) in the reference genome were annotated using RepeatMasker (https://www.repeatmasker.org/, accessed on 4 June 2025) and cpgIslandExt (https://genome.ucsc.edu/cgi-bin/hgTrackUi?g=cpgIslandExt, accessed on 4 June 2025), respectively.

Bismark (v0.24.0; https://www.bioinformatics.babraham.ac.uk/projects/bismark/, accessed on 4 June 2025) was employed for alignment of the bisulfite-treated reads. Briefly, both the reference genome and the clean reads were subjected to in silico C-to-T and G-to-A conversion. The converted reads were aligned to the converted genome (GCF_011004845.1) using bowtie2 (http://bowtie-bio.sourceforge.net/bowtie2/index.shtml; with parameters: -X 700 --dovetail; accessed on 4 June 2025) to identify the best unique match. The alignment coordinates were then transformed back to the original genome to determine the methylation status and precise genomic position of each cytosine. PCR duplicates, defined as reads aligning to the identical genomic start and end positions, were removed to mitigate amplification bias. Sequencing depth and genome coverage were calculated post-deduplication.

### 2.6. Methylation Analysis and Differential Methylation

Methylation calls were extracted using bismark_methylation_extractor (https://www.repeatmasker.org/; with the --no_overlap option to avoid double-counting overlapping reads in paired-end data; accessed on 4 June 2025) and visualized in IGV (https://igv.org/, accessed on 4 June 2025) after conversion to bigWig format. The bisulfite conversion efficiency was estimated based on the conversion rate of cytosines in the spiked-in lambda DNA genome. To identify methylated cytosines, a binomial test was performed for each cytosine position, using the number of methylated reads (mC), total coverage (mC + unmethylated C), and the non-conversion rate (1—bisulfite conversion efficiency) as parameters. Sites with an FDR-adjusted *p*-value < 0.05 were defined as significantly methylated.

To assess genome-wide methylation levels, the genome was tiled into non-overlapping 10-kb bins, and the average methylation level (ML) was calculated for each bin as (mC/(mC + umC)). Differentially methylated regions (DMRs) were identified using the DSS package (v2.12.0; https://bioconductor.org/packages/release/bioc/html/DSS.html, accessed on 4 June 2025), which employs a Bayesian hierarchical model to account for biological variation.

### 2.7. Functional Enrichment Analysis

Genes associated with the identified DMRs (i.e., those with DMRs in their promoter or structural gene) were subjected to functional enrichment analysis. Gene Ontology (GO) term enrichment was performed using the R package GOseq (v1.44.0; https://bioconductor.org/packages/release/bioc/html/goseq.html, accessed on 4 June 2025), which corrects for gene length bias, with a corrected *p*-value < 0.05 considered significant. KEGG pathway enrichment analysis was conducted using KOBAS software (v3.0; http://kobas.cbi.pku.edu.cn/kobas3/, accessed on 4 June 2025).

### 2.8. Methylation Validation of DMRs by Bisulfite Sequencing PCR (BSP)

Three DMRs with significant differences were randomly selected for methylation validation using bisulfite sequencing PCR (BSP). Primers for the DMRs were designed using the online software Methprimer 2.0 (http://www.urogene.org/cgi-bin/methprimer/methprimer.cgi, accessed on 4 June 2025). For the *ccdc180* gene, the primers were as follows: forward, TATTTTAGAGGGGGTAGAGGTGTTA; reverse, TCCTAAAACTACACCATTAACCTCC. For *ZNF501*, the primers were as follows: forward, TGTTGGAGTAATTTAGATTAGGTTTAGTG; reverse, CAAAATAACTAATAACTTCCCACACAAC. For *tshz1*, the primers were as follows: forward, GTTTGGAAAATTGGGTGATAGGT; reverse, TCTACTAATAACAATAAAAACAATCCACTC. To validate WGBS findings, bisulfite sequencing PCR (BSP) was conducted on the same genomic DNA using the EZ DNA Methylation-Gold™ Kit (Zymo Research). PCR was performed in a 30 μL system containing 5 μL of SybrGreen qPCR Master Mix (TransGen Biotech, Beijing, China), 1 μL of each primer (10 μM), 21 μL of ddH_2_O, and 2 μL of bisulfite-converted DNA. The protocol included initial denaturation (95 °C, 10 min) and 40 cycles of 94 °C for 30 s, 55 °C for 30 s, and 72 °C for 40 s. Amplified products were purified, cloned into the pMD19-T vector (TaKaRa Bio Inc., Shiga, Japan), and transformed into *E. coli* DH5α cells (TransGen Biotech, Beijing, China). A total of 10 positive clones per target were sequenced. The sequencing results were analyzed using DNAStar software (v17.0; https://www.dnastar.com/, accessed on 4 June 2025).

### 2.9. Statistical Analysis

Statistical analysis of the data was performed using IBM SPSS 27.0 (SPSS Inc., Chicago, IL, USA). Independent-samples *t*-tests were used for intergroup comparisons, with *p* < 0.05 indicating a statistically significant difference.

## 3. Results

### 3.1. Sex Identification

**Figure 1:**
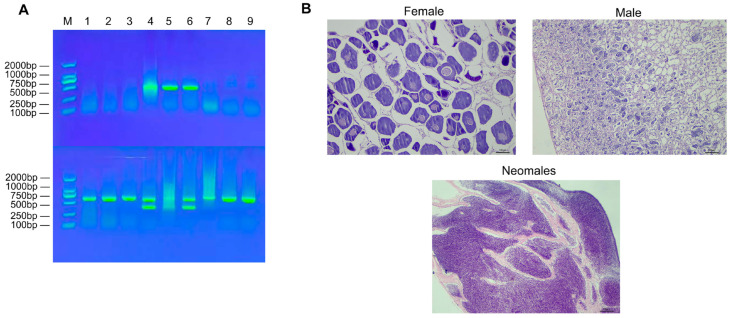
Schematic diagram for identification of genetic sex and physiological sex in *E. lucius*. (**A**) 1% agarose gel electrophoresis for male and female genotyping of *E. lucius*. Note: M: DNA marker; Upper row: 1–3: females; 4–6: males; 7–9: neomales.; Lower row: duplicate detection of the samples in the upper row. (**B**) Gonad sections of female, male, and neomale fish.

The genetic sex of all experimental fish was determined using laboratory-developed sex-specific molecular markers. Briefly, genomic DNA extracted from fin clips was amplified by PCR. Analysis by agarose gel electrophoresis clearly differentiated the genotypes (Figure 1A). Using primer set 4, a single 606-bp band was amplified in genetic females, while two bands (606 bp and 320 bp) were amplified in genetic males. Conversely, the Amh primer set produced a single band of 500 bp exclusively in genetic males, with no amplification in females. To confirm the phenotypic sex, gonadal histology was examined via paraffin sectioning (Figure 1B). Individuals that passed this dual verification—showing concordance between genetic and phenotypic sex—were definitively classified as females, males, or neomales (genetically female but phenotypically male) and used in subsequent experiments.

### 3.2. Overview of DNA Methylation

Table 1 presents the statistical results of Whole-Genome Bisulfite Sequencing (WGBS) performed on gonadal samples from different sexes of *E. lucius*. A total of nine sequencing samples were divided into three groups, with three biological replicates in each group. These samples collectively generated 410.16 Gb of raw sequences; after filtering, 361.48 Gb of high-quality sequence reads were finally obtained. These data were aligned to the *E. lucius* genome, with an average unique mapping rate of 86%. All subsequent bioinformatics analyses were based on these high-quality sequence reads. The bisulfite conversion rate of each sample exceeded 99.6%, and the average sequencing depth per sample was 30×.

A total of approximately 3745 million cytosine sites were identified in the genome, among which mCs accounted for an average of 7.10% (Table 2). Among all cytosine site types, the methylation rate of CG sites was the highest, representing over 80% of total mCs, followed by CHG and CHH sites. It can thus be concluded that genomic methylation in individuals of all sexes primarily occurs at CG sites. The statistical results revealed that the number of 5mCs was relatively small compared to the total number of cytosines in the whole genome. Among the three groups, the genomic DNA methylation level of neomales was higher than that of females and similar to that of males. The genomic DNA methylation levels of the nine samples ranged from 6.42% to 7.47%. Additionally, the statistical results indicated that 5mCs had the highest proportion in CG sequences, while their proportions in CHG and CHH sequences were relatively low, with average proportions of 0.49% and 0.49% across the nine samples, respectively (Table 2). Therefore, subsequent analyses focused solely on CpG sites.

**Figure 2:**
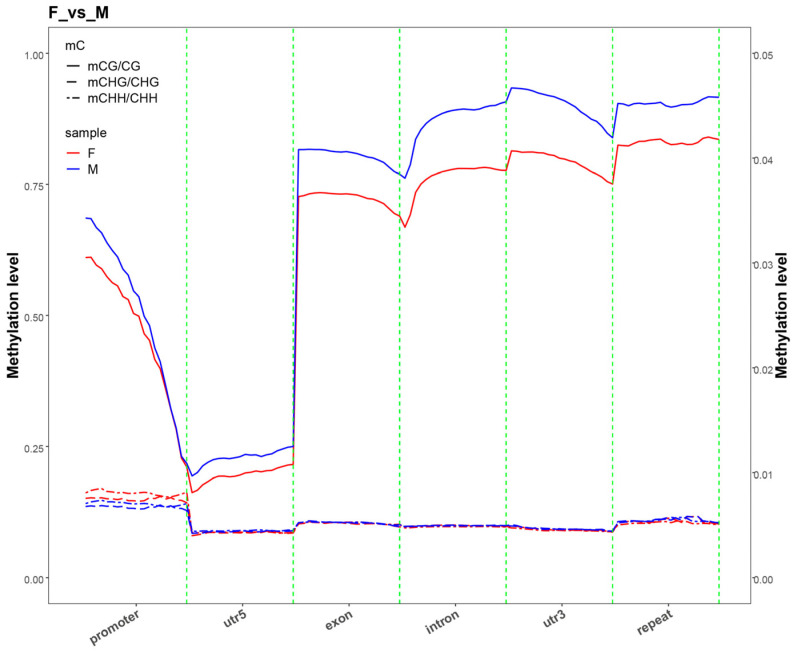
DNA methylation landscape across core genic elements in female (F) and male (M) *E. lucius.* Note: The average cytosine methylation levels were analyzed in key genic features, including promoters, exons, introns, and downstream regions. The methylation level for each context was calculated as mC/(mC + uC) and is presented as the mean ± SD.

To analyze differences in methylation levels across various genomic functional regions among different sex combinations, we examined methylation levels in regions including promoters, exons, introns, CGIs (CGIs, genomic regions enriched with CpG dinucleotides that are pivotal for gene regulation), CGI shores (the flanking regions up to 2 kb adjacent to CGIs), and repeat regions. From a global methylation perspective, the total cytosine methylation rate (MeanC%) was significantly lower in females than in males and neomales (*p* < 0.05). Notably, the CpG site methylation rate (MeanCG%) exhibited a more pronounced sex difference, with females showing extremely significantly lower levels compared to males and neomales (*p* < 0.001), whereas no significant differences were observed between males and neomales for either indicator. By contrast, the CHG and CHH site methylation rates (MeanCHG%/MeanCHH%) did not differ significantly among the three groups (*p* > 0.05). The results showed that, in methylation patterns dominated by the CG sequence context, the methylation levels of neomales in regions such as exons, introns, and 3′ UTR were overall significantly higher than those of females, with clear differences between the two groups. This observation is consistent with the general rule that mCG methylation levels in males are higher than those in females under the CG context, reflecting that neomales and females exhibit significant differentiation in CG methylation regulation within core functional regions of the structural gene. Additionally, neomales and males showed no significant inter-group differences in methylation levels in the above functional regions. For methylation related to CHG and CHH sequence contexts, all sex combinations exhibited no significant inter-group differences in methylation levels within these functional regions (*p* > 0.05). These results indicated that there were no significant differences in methylation levels among the groups within the same functional region or transcriptional element (Figure 2 and Figure S1).

**Figure 3:**
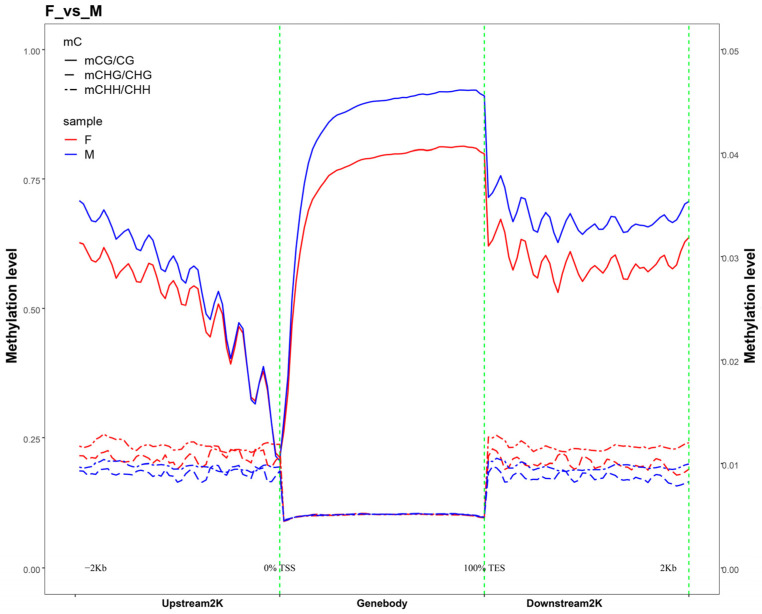
Distribution of methylation levels in the upstream and downstream 2 K regions of genes. Note: The plot depicts the average DNA methylation levels within 2 kilobases (kbs) up- and downstream of all annotated genes. The transcription start site (TSS) and transcription termination site (TTS) are marked with dashed lines for reference.

CpG sequences exhibited significant differences in methylation levels across various genomic functional elements: methylation levels decreased significantly in the 2-kb region upstream of genes; were the lowest in the 5′ UTR; reached the highest in coding sequences, introns, and 3′ UTR; and decreased slightly but remained relatively high in the 2-kb region downstream of genes. By contrast, CHG and CHH sequences showed very low methylation levels across all gene functional elements with no clear variation. Further comparison of methylation levels in the 2-kb regions upstream and downstream of the genes (Figure 3 and Figure S2) revealed that, in the CG context, the methylation level was lowest at the transcription start site (TSS) and highest at the transcription termination site (TES), exhibiting a gradual upward trend from TSS to TES, whereas in the CHG and CHH contexts, the methylation levels were extremely low across all regions.

### 3.3. DMR Analysis

Genome-wide differential methylation analysis identified a total of 18,374 DMRs in the CG context, along with 254 DMRs in CHG and 584 DMRs in CHH contexts. Of these, 11,784 regions (11,277 CG, 145 CHG, 362 CHH) were hypermethylated, and 7428 were hypomethylated (7097 CG, 109 CHG, 222 CHH). Annotation of these DMRs revealed their distribution across various genomic elements. As illustrated in Figure S3 and Table 3, the majority were enriched in CpG islands (CGIs), with substantial numbers also located in introns, exons, and promoter regions, while fewer were found in 5′ UTRs and repetitive elements.

Pairwise comparisons among male, female, and neomale fish were conducted using a 10-kb sliding window method to detect differentially methylated regions (DMRs). The identified DMRs were anchored in whole-gene regions (spanning from the transcription start site, TSS, to the transcription end site, TES) and promoter regions, and genes unique to the CG methylation context were predominant in all gender combinations.

**Figure 4:**
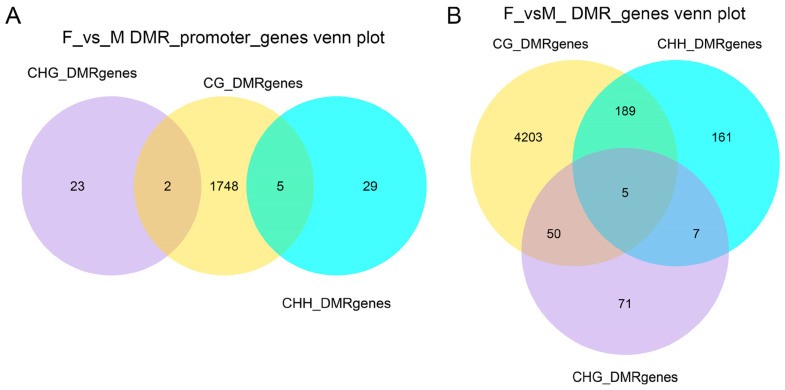
Venn diagrams of genes associated with differentially methylated regions (DMRs). (**A**) Genes with DMRs located anywhere across the whole structural gene (from TSS to TES). (**B**) Genes with DMRs specifically located within their promoter regions.

For promoter regions (Figure 4A and Figure S4A), the F_vs._M comparison revealed 23, 1748, and 29 DMRs unique to the CHG, CG, and CHH contexts, respectively; 2 DMRs were shared between the CG and CHG contexts; and 5 DMRs were shared between the CG and CHH contexts. The M_vs._NM comparison identified 13, 1003, and 37 DMRs unique to the CHG, CG, and CHH contexts, respectively, with 2 DMRs shared between the CG and CHH contexts. For the F_vs._NM comparison, there were 14, 1758, and 12 DMRs unique to the CHG, CG, and CHH contexts, respectively; 1 gene was shared between the CG and CHG contexts, and 4 DMRs were shared between the CG and CHH contexts.

For whole-gene regions (Figure 4B and Figure S4B), the F_vs._M comparison demonstrated 4203, 71, and 161 DMRs unique to the CG, CHG, and CHH contexts, respectively; 50 DMRs were shared between the CG and CHG contexts, 189 DMRs between the CG and CHH contexts, 7 DMRs between the CHH and CHG contexts, and 5 DMRs were shared among all three contexts. The F_vs._NM comparison found 4188, 77, and 170 DMRs unique to the CG, CHG, and CHH contexts, respectively; 51 DMRs were shared between the CG and CHG contexts, 167 DMRs between the CG and CHH contexts, 9 DMRs between the CHH and CHG contexts, and 16 DMRs were shared among all three contexts. For the M_vs._NM comparison, there were 2898, 101, and 220 DMRs unique to the CG, CHG, and CHH contexts, respectively; 34 DMRs were shared between the CG and CHG contexts, 126 DMRs between the CG and CHH contexts, 3 DMRs between the CHH and CHG contexts, and 9 DMRs were shared among all three contexts.

### 3.4. Functional Enrichment Analysis of DMGs

**Figure 5:**
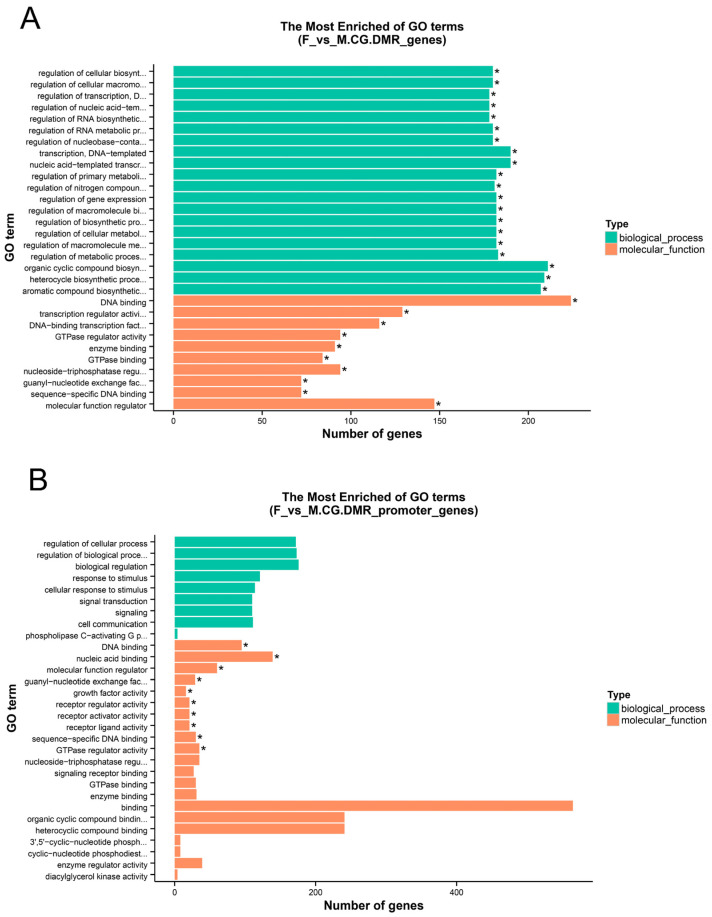
Gene Ontology (GO) enrichment analysis. * Corrected *p*-value < 0.05. (**A**) F vs. M. CG.DMR_genes; (**B**) F vs. M. CG.DMR_promoter_genes.

GO and KEGG enrichment analyses were performed on the anchored genes. Since most DMRs belong to the CG context, enrichment analysis was conducted only for CG methylation. Based on the GO database, the F vs. M comparison revealed 30 significantly enriched terms each in the structural gene (TSS to TES) and promoter regions (corrected *p*-value < 0.05). The significantly enriched GO terms primarily included DNA binding, nucleic acid binding, and sequence-specific DNA binding. For the F vs. NM comparison, significant enrichment was observed for 30 GO terms in the structural gene and 2 terms in the promoter region (corrected *p*-value < 0.05). The top 30 terms across all comparisons, ranked by corrected *p*-value, are presented in Figure 5 and Table S1. The most significantly enriched GO terms, which were primarily associated with DNA binding and nucleic acid binding, are presented in ascending order of corrected *p*-value (Figure S5 and Table S2). In the M vs. NM comparison, 27 GO terms were found to be significantly enriched within the structural gene region, while no significant GO term enrichment was detected in the promoter region (corrected *p*-value < 0.05). The significantly enriched GO terms included cell adhesion, membrane, and transmembrane transporter activity. The top 30 GO terms, ranked in ascending order by corrected *p*-value, are shown (Figure S5 and Table S3).

**Figure 6:**
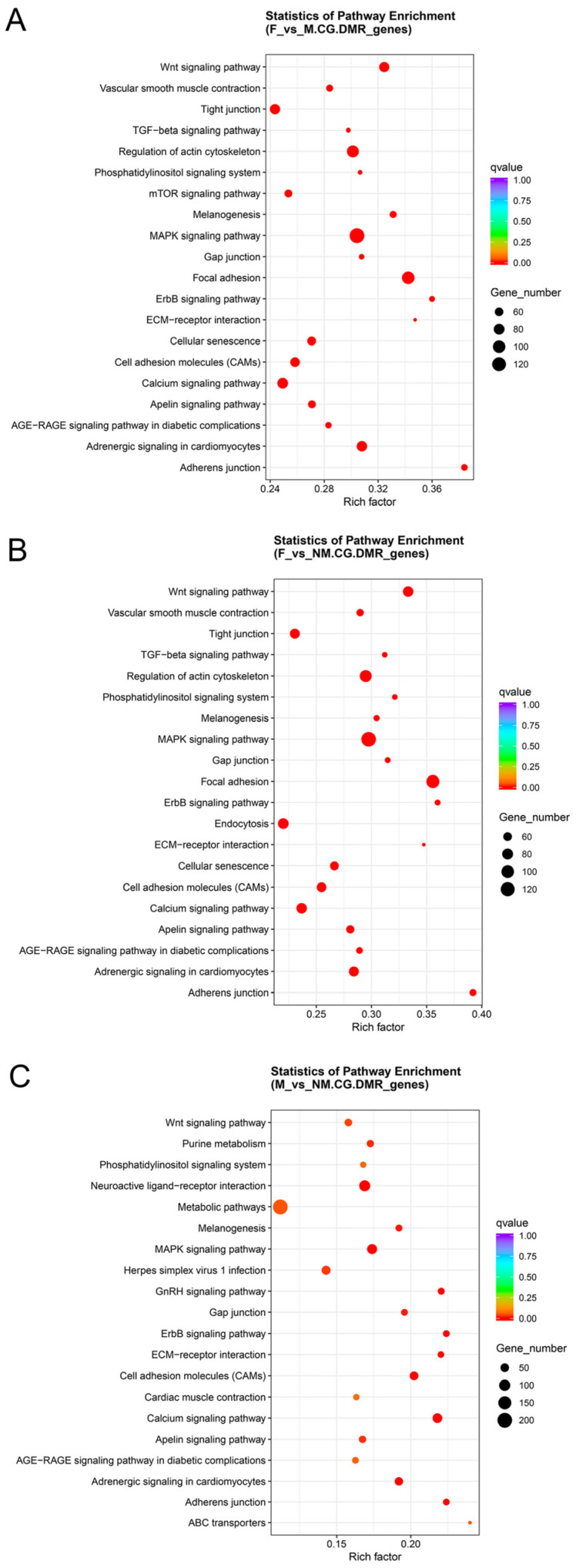
Scatter plots of KEGG pathway enrichment analysis for CG.DMR_genes in *E. lucius* among three sex combinations. Note: (**A**) female vs. male (F vs. M); (**B**) female vs. neomale (F vs. NM); (**C**) male vs. neomale (M vs. NM).

KEGG enrichment analysis for the F vs. M comparison revealed 157 and 132 significantly enriched pathways in the structural gene (from TSS to TES) and promoter regions, respectively (corrected *p*-value < 0.01). The top 20 most significant pathways are displayed in Figure 6 and Table S4. Pathways consistently enriched in both the structural gene and promoter regions included the MAPK signaling pathway, Wnt signaling pathway, Notch signaling pathway, progesterone-mediated oocyte maturation, and oocyte meiosis. In the F vs. NM comparison, 153 pathways were significantly enriched in the structural gene region (corrected *p*-value < 0.01), while 132 pathways were significantly enriched in the promoter region (corrected *p*-value < 0.01). The top 20 most significantly enriched pathways are displayed in Figure S5 and Table S4. The pathways co-enriched in both the structural gene and promoter regions were identical to those identified in the F vs. M comparison, with the exception of endocytosis and the mTOR signaling pathway. In the M vs. NM comparison, there were 156 and 126 significantly enriched pathways in the structural gene and promoter regions, respectively (corrected *p*-value < 0.01). Figure S5 and Table S4 present the 20 pathways with the highest enrichment levels. Pathways co-enriched in both regions included the Wnt and GnRH signaling pathways.

Combining the localization of whole-genome DMRs with functional analysis of DMGs, most of the genes enriched in GO and KEGG analyses were located in intron regions. Further screening was conducted on the genes significantly enriched in GO and KEGG, revealing that *Rspo1*, *hsd11b2*, *CYP27A1,* and *smad3* may be involved in the sex determination and differentiation of *E. lucius*.

### 3.5. Verification of Differentially Methylated Genes

**Figure 7:**
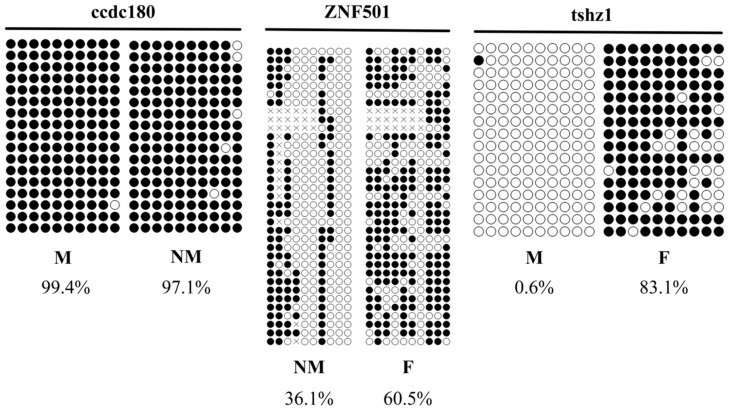
Validation of WGBS data for *E. lucius* by bisulfite sequencing PCR. F: female; M: male; NM: neomale. Note: Each horizontal strand represents a sequenced clone, each circle represents a CpG site, and white and black circles represent unmethylated and methylated CpG sites, respectively.

Differentially methylated genes (DMGs) were randomly selected for validation using genome-wide methylation data. Three genes (*ccdc180*, *ZNF501*, and *tshz1*) were selected for BSP (bisulfite sequencing PCR) validation. The methylation level of *ccdc180* DMR in the M group was significantly lower than that in the NM group, with a methylation difference of approximately 18.0%. The methylation level of *ZNF501* DMR in the F group was significantly higher than that in the NM group (*p* < 0.05), with a methylation difference of about 50.3%. The methylation level of *tshz1* DMR in the F group was significantly higher than that in the M group, with a methylation difference of approximately 82.9%. In the BSP sequencing results, the trend in methylation levels was consistent with the WGBS data (Table 4 and Figure 7).

## 4. Discussion

In this study, Whole-Genome Bisulfite Sequencing (WGBS) on gonadal tissue samples from females, males, and neomales of *E. lucius* was performed. A total of 410.16 Gb of raw reads and 361.48 Gb of clean reads were obtained; the average unique mapping rate reached 86%, indicating high sequencing quality and laying a solid foundation for subsequent analyses. The DNA methylation levels at CpG sites in all three groups exceeded 73%, while the methylation levels at CHG and CHH sites were both below 0.52%. This suggests that methylated CpG (mCpG) is the dominant DNA methylation pattern in the gonadal tissues of *E. lucius*. This finding is consistent with the mCpG-dominant DNA methylation pattern observed in the tissues of other fish species (e.g., T. *rubripes*) [24]. Furthermore, the results of this study showed that the number of cytosine methylations at CpG sites is higher than that at CHG and CHH sites, which is consistent with the conclusions drawn from studies on sheep ovaries and bovine sperm [26,27]. This CpG-specific characteristic pattern of DNA methylation stems from the preferential action of DNA methyltransferases (DNMTs) on CpG dinucleotides. Consequently, mCpG is abundant in the genomes of vertebrates, particularly in promoter regions, structural gene regions, and repetitive elements; it plays a crucial role in the regulation of gene expression and transcriptional silencing [28,29].

To elucidate how and why the expression of these key genes diverges among females, males, and neomales, we propose that sex-specific DNA methylation acts as a primary regulatory switch. This epigenetic mechanism orchestrates a transcriptional reprogramming that directs gonadal fate. Specifically, the differential methylation we observed in the regulatory regions of *Rspo1*, *smad3*, *hsd11b2*, and *CYP27A1* creates a coordinated expression landscape that reinforces either the female or male developmental pathway. The most profound reprogramming is evident in neomales, where a male-like methylation pattern is superimposed upon a female genetic background, effectively silencing ovarian-promoting genes and activating testicular-promoting ones, thereby overriding the original genetic sex and leading to the full manifestation of a male phenotype.

In the field of animal breeding, changes in DNA methylation patterns are closely associated with sex determination and sex differentiation processes. By analyzing the methylation patterns of individuals, it is possible to identify key genes and regulatory pathways related to sex determination and sex differentiation, which is of great significance for the targeted breeding of monosex aquaculture populations, optimization of sex-control production strategies, and development of sex-related molecular markers. We identified four differentially methylated genes (DMGs) associated with sex determination and differentiation processes in the gonadal tissues of females, males, and neomales of *E. lucius*, namely, *Rspo1*, *hsd11b2*, *CYP27A1*, and *smad3*. R-spondin 1 (*Rspo1*) is an activator of the Wnt/β-catenin signaling pathway and has been identified as a novel key factor involved in sex and ovarian differentiation [30]. Loss of *Rspo1* in Nile tilapia (*O. niloticus*) leads to oocyte defects in XX fish and spermatocyte defects in XY fish, indicating that the signaling pathway activated by *Rspo1* is involved in the development of both ovaries and testes [31]. Enhanced *Rspo1* function induces feminization of XY medaka (*Oryzias latipes*), and Rspo1-OV-XY females are fertile and produce viable offspring [32]. These observations suggest that *Rspo1* is a crucial regulator of sex determination and development activation via the canonical Wnt/β-catenin signaling pathway. 11β-hydroxysteroid dehydrogenase 2 (*hsd11b2*) is a key enzyme involved in the metabolic regulation of steroid hormones, and plays a vital role particularly in the processes of sex determination and differentiation. It influences the sex development pathway of fishes by regulating the synthesis of cortisol and 11-ketotestosterone (11-KT) [33]. For instance, in the spotted knifejaw (*Oplegnathus punctatus*), the activity of *hsd11b2* is directly associated with the production of 11-KT—a major androgen in fishes that is essential for male differentiation [34,35]. Furthermore, the expression of *hsd11b2* is significantly higher in male gonads than in female gonads, which further supports its importance in male development [35,36]. Cytochrome P450 26A1 (*CYP26A1*) is a key retinoic acid (RA) metabolizing enzyme that plays a critical role in the processes of sex determination and differentiation in vertebrates. Its function is mainly reflected in regulating the degradation of retinoic acid, thereby affecting gonadal development and the fate of germ cells. Studies on the Chinese soft-shelled turtle (*Pelodiscus sinensis*) have revealed that the expression of *CYP26A1* in the gonads of male embryos is significantly higher than that in female embryos (*p* < 0.05), indicating its important role in male gonadal development. The coding sequence of *CYP26A1* contains a 1485-bp open reading frame (ORF) encoding 494 amino acids, and its functional domain is associated with retinoic acid degradation. These results suggest that *CYP26A1* may play a key role in promoting male gonadal development [37]. In addition, in *D. rerio*, the expression of *CYP26A1* exhibits sexual dimorphism during gonadal development: its expression is upregulated in the testes, whereas no similar phenomenon is observed in the ovaries. Studies have shown that *CYP26A1* may affect the fate of germ cells by inhibiting the initiation of meiosis [38]. In studies on the sea cucumber (*Apostichopus japonicus*), *Smad3* has been identified as a key regulatory gene in the processes of sex differentiation and gonadal development. Research indicates that *Smad3* affects the synthesis of sex hormones by participating in the AMPK and TGF-β signaling pathways, thereby regulating gonadal development and function [39].

*Rspo1* is hypomethylated in F vs. M and F vs. NM but shows no change in M vs. NM. The promoter region of the *Rspo1* gene is hypermethylated, indicating that *Rspo1* is specifically activated in females to promote ovarian development. By contrast, its status is similar in males and neomales, where it is likely inhibited, leading to the shutdown or downregulation of its expression. This essentially cuts off one of the primary signaling pathways that maintain female identity, clearing the way for ovarian regression and testicular initiation. *Smad3* is hypomethylated in F vs. M and F vs. NM but hypermethylated in M vs. NM. In female fish, the hypomethylation of *smad3* results in its continuous activation, which may maintain ovarian development or inhibit the male pathway via the TGF-β signaling pathway. In male fish, the hypermethylation of *smad3* leads to its silencing, potentially allowing the testicular development program to proceed smoothly. In neomales, the methylation level of *smad3* is intermediate between that of females and males, reflecting its characteristic of having a genetic female background but a phenotypic male identity. Both *hsd11b2* and *CYP27A1* are hypomethylated in F vs. M, F vs. NM, and M vs. NM, suggesting that these genes are most active in females and least active in neomales. This may imply that they are involved in female-specific metabolic pathways or that their hypomethylation is part of the maintenance of the female phenotype. In neomales, the key female gene *Rspo1* is hypermethylated and silenced, which inhibits the Wnt/β-catenin pathway and blocks the ovarian development route. The regulatory factor *smad3* is partially silenced, weakening the TGF-β pathway signal and further promoting the differentiation of germ cells toward spermatogonia. Meanwhile, *hsd11b2* and *CYP27A1* maintain or enhance their expression through hypomethylation, optimizing the internal androgen environment and facilitating the full manifestation of the male phenotype.

Our KEGG enrichment analysis revealed strong similarity between the F vs. M and F vs. NM comparisons, with the top 20 enriched pathways showing substantial overlap. This indicates that neomales exhibit a molecular profile, particularly in key signaling pathways, that closely resembles that of genetic males, supporting the use of neomales as a model for studying sex reversal. Notably, conserved pathways such as MAPK, Wnt, and TGF-beta signaling were enriched in both comparisons, all of which are well-established regulators of vertebrate sex determination. This underscores their central role in driving male-specific development, regardless of whether the male phenotype arises from genetic or environmental origins. The shared enrichment of “Oocyte meiosis” and “Progesterone-mediated oocyte maturation” likely reflects the suppression of female gametogenesis in both males and neomales, indicating a comprehensive shift away from the ovarian fate.

Some subtle differences were observed: “Endocytosis,” which is critical for receptor signaling and cellular homeostasis, was uniquely enriched in F vs. NM. This may suggest altered turnover of receptors involved in sex maintenance or stress response, potentially representing a distinctive adaptation during sex reversal. Meanwhile, the “mTOR signaling pathway,” linked to nutrient sensing and growth processes, was specific to F vs. M, possibly reflecting developmental differences between natural and induced male phenotypes.

## 5. Conclusions

We conducted whole-genome DNA methylation sequencing analysis on the gonadal tissues of *E. lucius* and successfully identified four key differentially methylated genes (DMGs) closely associated with sex determination and sex differentiation processes, namely, *Rspo1, hsd11b2, CYP27A1,* and *smad3*. These research findings provide crucial gene targets and a theoretical framework for elucidating the epigenetic mechanisms of sex differentiation in *E. lucius*. Furthermore, this study lays a foundation for developing precise sex-control technologies, such as directing gonadal development and optimizing sex ratios in farmed populations, which will support the sustainable growth of the *E. lucius* aquaculture industry.

## Figures and Tables

**Table 1:** Methylation sequencing data for males, females and neomales of *E. lucius*. Note: F: female fish, M: male fish, NM: neomale fish. The numbers following the group codes (e.g., F1, M2, NM3) denote individual biological replicates (*n* = 3 per group).

Sample	NM1	NM2	NM3	F1	F2	F3	M1	M2	M3
Raw reads	146,386,382	143,070,174	147,693,243	182,910,368	131,255,545	141,324,771	162,368,975	145,235,514	166,940,890
Clean reads	140,347,706	136,438,226	141,696,961	174,884,508	125,792,497	135,484,511	155,349,601	139,302,719	159,513,154
Mapped reads	119,970,522	116,879,994	121,939,108	150,052,149	107,683,670	116,419,144	131,476,036	119,840,015	135,549,748
Unique mapping rate (%)	79.66	80.26	80.46	80.47	80.22	80.54	79.17	80.14	79.36
Duplication rate (%)	37.26	36.71	34.21	36.71	38.35	37.41	34.77	34.06	30.68
Q20 (%)	99.41	99.40	99.40	99.42	99.43	99.44	99.40	99.42	99.39
Q30 (%)	96.46	96.41	96.41	96.47	96.55	96.58	96.50	96.48	96.48
GC (%)	22.69	22.68	23.07	22.46	22.28	22.90	22.53	22.96	22.67
BS conversion rate (%)	99.670	99.663	99.665	99.655	99.623	99.660	99.650	99.647	99.614

**Table 2:** Methylation sequencing data for males, females and neomales of *E. lucius*. Note: C_covgMean: mean coverage depth of cytosine sites; C (Mb): total number of cytosine sites (unit: megabase equivalent, used for site quantity statistics); CG: number of CpG dinucleotide sites; CHG: number of cytosine-purine-guanine sites; CHH: number of cytosine-purine-purine sites (H = A/T/C), MeanC (%): methylation ratio of total cytosine sites; MeanCG (%): methylation ratio of CpG sites; MeanCHG (%): methylation ratio of CHG sites; MeanCHH (%): methylation ratio of CHH sites.

Sample	NM1	NM2	NM3	F1	F2	F3	M1	M2	M3
C_covgMean	9.1	9.0	9.6	11.5	8.0	8.7	10.5	9.3	11.3
C (Mb)	3539.1	3491.1	3705.6	4450.8	3111.2	3360.3	4062.8	3622.2	4361.6
CG (Mb)	292.1	287.1	314.4	365.5	253.0	284.1	328.6	304.8	357.8
CHG (Mb)	769.8	758.0	817.3	965.8	670.6	734.4	877.1	796.5	945.5
CHH (Mb)	2477.1	2446.0	2574.0	3119.6	2187.6	2338.8	2857.2	2520.8	3058.3
MeanC (%)	7.36	7.32	7.55	6.54	6.42	6.69	7.22	7.47	7.36
MeanCG (%)	83.79	83.67	84.01	74.14	73.15	74.05	83.48	83.55	83.96
MeanCHG (%)	0.48	0.48	0.47	0.50	0.52	0.48	0.51	0.48	0.52
MeanCHH (%)	0.48	0.48	0.46	0.49	0.51	0.47	0.51	0.47	0.51

**Table 3:** Distribution of DMRs in CG, CHG, and CHH. Note: CG, CpG dinucleotide context; CHG, cytosine–purine–guanine context; CHH, cytosine–purine–guanine context (where H = A, T, or C); Hyper, hypermethylated DMRs; Hypo, hypomethylated DMRs.

Gene_Region	CG		CHG		CHG	
	Hyper	Hypo	Hyper	Hypo	Hyper	Hypo
CGI	1913	3438	28	31	45	37
CGI_shore	3379	5818	76	114	178	215
promoter	2146	2552	19	25	36	48
TSS	756	832	2	1	3	5
utr5	984	2022	3	2	17	12
exon	2572	5018	17	34	99	119
intron	6564	11,522	166	231	504	578
utr3	567	645	5	4	9	15
TES	348	326			2	2
repeat	2441	7637	131	204	505	566
other_region	2712	2389	56	66	86	104

**Table 4:** DMR methylation levels in the gonadal tissue of *E. lucius* WGBS.

Gene	Groups	Chr	Start	End	Position	Meth. diff
*ccdc180*	MvsNM	chr1	13,680,548	13,680,802	Exon	0.179
*ZNF501*	FvsNM	chr11	7,452,085	7,452,391	Exon	0.503
*tshz1*	FvsM	chr20	6,791,218	6,791,578	Exon	0.828

## Data Availability

The results of whole-genome methylation sequencing of *Esox lucius* have been deposited in the BioProject database of the National Center for Biotechnology Information (NCBI) with the accession number PRJNA1356644, and the public access URL is: https://www.ncbi.nlm.nih.gov/bioproject/PRJNA1356644 (accessed on 10 December 2025).

## Supplementary Materials

The following supporting information can be downloaded at: https://www.mdpi.com/article/10.3390/ani15243594/s1, Figure S1: Distribution of methylation levels on gene functional elements; Figure S2: Distribution of methylation levels in the upstream and downstream 2 K regions of genes; Figure S3: Distribution of DMRs in various functional genomic elements; Figure S4: Venn diagrams of genes anchored to DMRs; Figure S5: Bar charts of enriched GO terms; Table S1: Top GO terms in the FvsM; Table S2: Top GO terms in the FvsNM; Table S3: Top GO terms in the MvsNM; Table S4: The top KEGG pathways in the FvsM; Table S5: The top KEGG pathways in the FvsNM; Table S6: The top KEGG pathways in the MvsNM.
