# Supplementary material for: Whole-Genome Methylation Analysis of Female, Male, and Neomale Northern Pike (Esox lucius)
Source: Animals (Basel). 2025 Dec 15;15(24):3594. doi: 10.3390/ani15243594 (PMC12729703; doi:10.3390/ani15243594)
Supplement: Supplementary file 1 [file animals-15-03594-s001.zip › animals-3999276-supplementary.pdf]

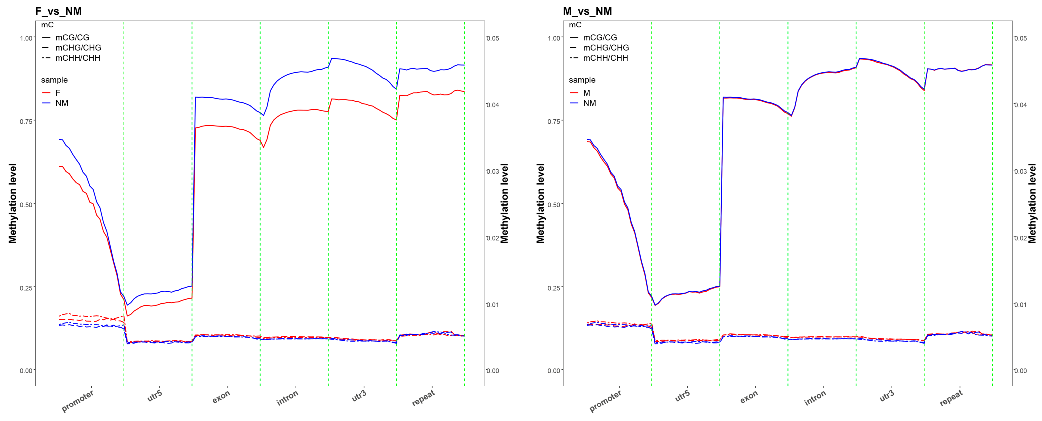

Figure S1 Distribution of methylation levels on gene functional elements. (A) F vs. NM; (B)M vs. NM.

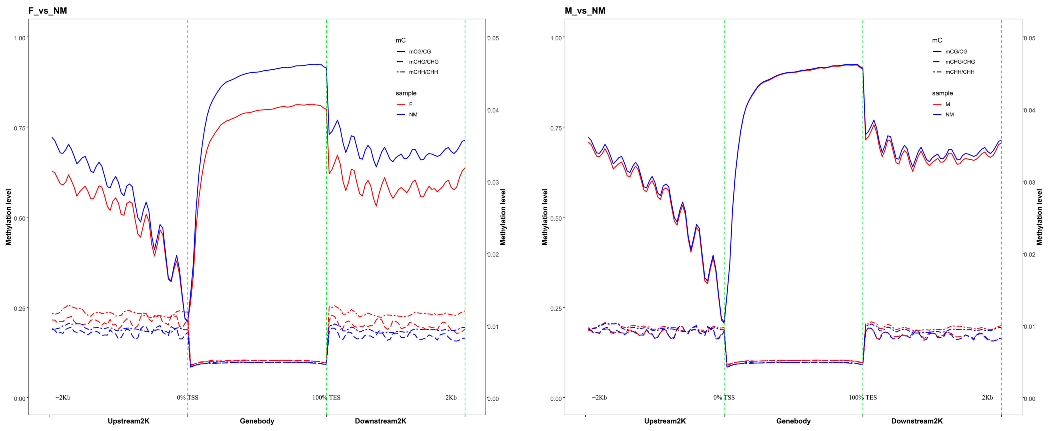

Figure S2. Distribution of methylation levels in the upstream and downstream 2 K regions of genes.(A) F vs. NM; (B) M vs. NM.

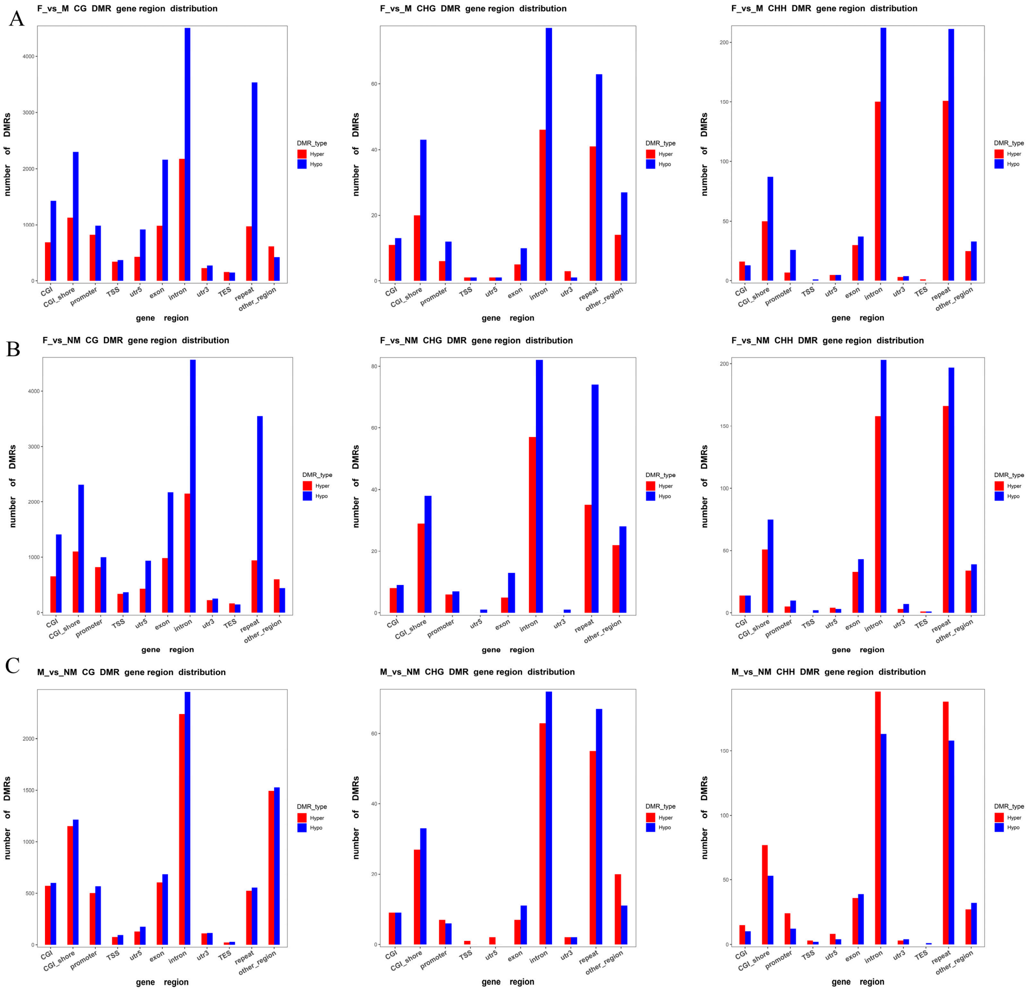

Figure S3. Distribution of DMRs in various functional genomic elements. (A) F vs. M; (B) F vs. NM; (C) M vs NM

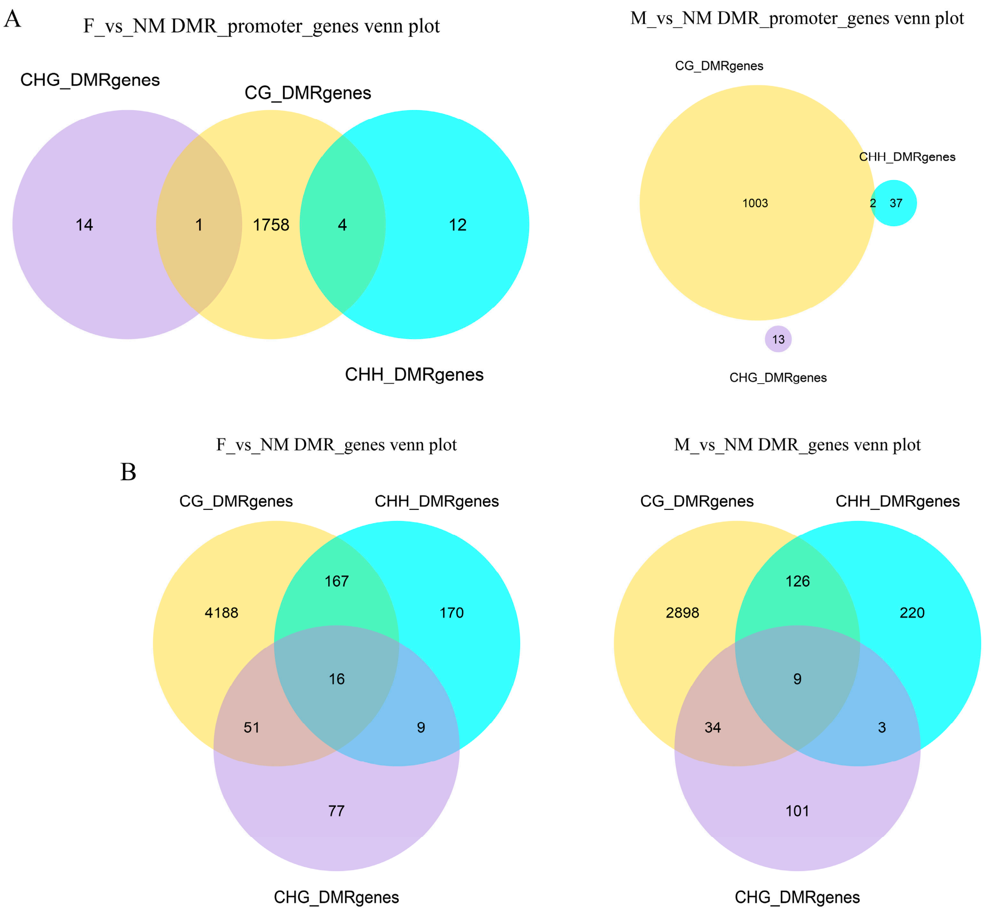

Figure S4. Venn diagrams of genes anchored to DMRs. (A) Anchored Genes; (B) genes related to anchored promoter regions.

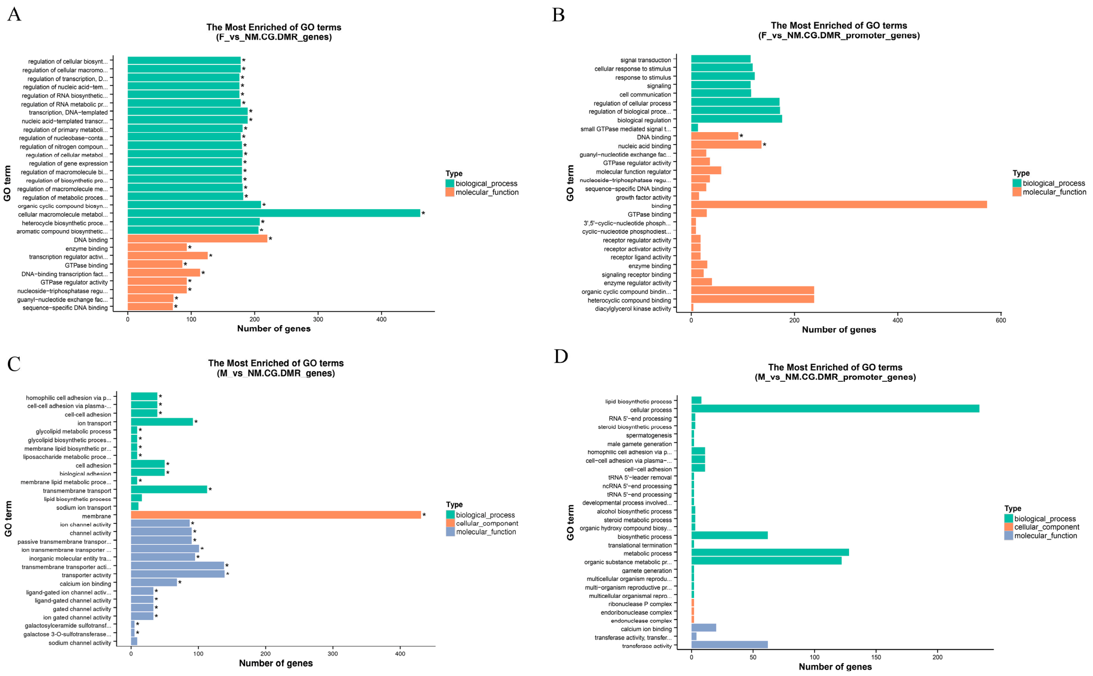

Figure S5. Bar charts of enriched GO terms. * Corrected p-value < 0.05. (A) F vs. NM. CG.DMR_genes; (B) F vs. NM. CG.DMR_promoter_genes; (C) M vs. NM. CG.DMR_genes; (D) M vs. NM. CG.DMR_genes.
